# From support to sustained engagement: internalization processes in experiential learning in tourism management education

**DOI:** 10.3389/fpsyg.2026.1836705

**Published:** 2026-05-14

**Authors:** Jingna Zhao, Qingqing Liu

**Affiliations:** 1 Graduate School of Tourism Management, Woosuk University, Wanju, Republic of Korea; 2 School of Tourism, Woosuk University, Wanju, Republic of Korea

**Keywords:** Education for Sustainable Development (ESD), psychological safety, student participation support, sustainable experiential learning, teacher instructional guidance, tourism management education

## Abstract

**Purpose:**

Grounded in the frameworks of Education for Sustainable Development (ESD) and Sustainable Development Goal 4.7 (SDG 4.7), this study explores how support mechanisms in experiential learning within tourism management influence students’ sustained engagement through processes of internalization.

**Methods:**

A qualitative research design was adopted. Guided by constructivist grounded theory, semi-structured interviews were conducted with tourism management teachers (*n* = 10) and students (*n* = 10) from two universities in Jeollabuk-do, South Korea. The data were analyzed through open coding, axial coding, and selective coding.

**Findings:**

The findings indicate that: (1) teacher instructional guidance demonstrates a dual-pathway structure consisting of structured guidance and reflective–emotional guidance, which provides students with action pathways and regulates uncertain learning contexts; (2) student participation support sustains the progression of learning through mechanisms of cognitive coordination and emotion regulation; and (3) during sustained engagement, these forms of support are gradually internalized, manifested in the formation of psychological safety, which further reinforces self-efficacy and participation interest, and ultimately transforms into stable positive psychological experiences, thereby supporting sustained engagement.

**Conclusion:**

From a process-oriented perspective, this study reveals the formation mechanism of support–internalization–sustained engagement in experiential learning within tourism management education.

## 1 Introduction

Within the framework of Sustainable Development Goal 4.7 (SDG 4.7) and Education for Sustainable Development (ESD), higher education has gradually shifted from the transmission of disciplinary knowledge toward fostering students’ professional competence, social responsibility, and sustainable development capacities in authentic contexts (Abo-Khalil, 2024; Höffken and Lazendic-Galloway, 2024; Holst et al., 2024). Tourism management education, due to its deep embeddedness in industrial practice, social interaction, and contextualized decision-making, has been increasingly regarded as an important arena for implementing ESD principles (Chen et al., 2022; Maguire et al., 2024). In particular, within experiential learning activities such as fieldwork, project-based learning, service-learning, and university–community collaboration, students are required to sustain participation, coordinate relationships, and engage in reflective learning under complex and uncertain conditions (Croft and Wang, 2025; Doğantan, 2023; Liang, 2021; Narciss and Alemdag, 2025). However, the implementation of experiential learning does not in itself necessarily lead to sustainable learning outcomes (Sundman et al., 2025; Zopiatis et al., 2021). Tourism management learning is typically characterized by open-ended tasks, rapidly changing situations, and multi-actor collaboration (Guo and Fryer, 2025), which may easily generate feelings of uncertainty, doubts about one’s capabilities, and emotional burden during participation (Bureau et al., 2021). Therefore, compared with the question of whether students participate in experiential learning, a more critical issue is whether the learning process can, under sustained support, be transformed into stable engagement, competence development, and positive psychological experiences (Annelin and Boström, 2024; Li and Fan, 2026). In this sense, sustainable experiential learning in tourism management should not be understood as a series of isolated instructional activities, but rather as a learning ecosystem formed through continuous interaction and support conditions (Annelin and Boström, 2024; Höffken and Lazendic-Galloway, 2024; Sundman et al., 2025).

Although existing studies have addressed experiential learning and sustainable tourism education, most have focused on outcome measurement or the analysis of single factors (Chen et al., 2022; Doğantan, 2023; Maguire et al., 2024). There remains a lack of fine-grained, process-oriented explanation of how teacher instructional guidance and student participation support jointly operate in the concrete learning process (Schutte et al., 2025). In particular, within the same learning context, relatively limited attention has been paid to how teachers support students through task scaffolding and feedback-oriented guidance, how students sustain learning progression through collaborative participation and peer interaction, and how these forms of support are understood by students and further associated with their sustained engagement and development (Li, 2025; Liu et al., 2023; Parmar et al., 2025; Senden et al., 2023; Wang et al., 2024). Accordingly, this study takes experiential learning in tourism management education as its context and explores the practices and functions of support mechanisms from the dual perspectives of teachers and students. Specifically, this study addresses the following research questions:

RQ1: In experiential learning within tourism management education, how is teacher instructional guidance enacted in practice, and how does it support students’ sustained engagement and competence development?

RQ2: During the learning process, how do students experience and construct student participation support, and how is learning progression sustained through multi-actor interaction?

RQ3: How are teacher instructional guidance and student participation support interconnected within the same learning context, and how do they influence students’ sustained engagement and learning experiences through mechanisms such as psychological safety and self-efficacy?

Through a process-oriented analysis of these questions, this study aims to reveal the formation mechanism of a sustainable experiential learning ecosystem in tourism management education and to provide more explanatory qualitative evidence for research on learning processes and support systems within the ESD framework (Holst et al., 2024; Schutte et al., 2025; Sundman et al., 2025).

## 2 Theoretical framework

### 2.1 Experiential learning in tourism management from an ESD perspective

Within the frameworks of Education for Sustainable Development (ESD) and Sustainable Development Goal 4.7 (SDG 4.7), higher education increasingly emphasizes students’ sustained engagement, sense of responsibility, and the development of integrated competencies in authentic contexts (Abo-Khalil, 2024; Höffken and Lazendic-Galloway, 2024).

Experiential learning theory provides a foundational framework for learning through experience. (Kolb, 1984) describes experiential learning as a cycle of concrete experience, reflective observation, abstract conceptualization, and active experimentation. However, it does not explicitly explain sustained engagement in complex learning contexts. Therefore, this study extends Kolb’s model by examining how support mechanisms foster sustained engagement through internalization.

This requirement is particularly salient in tourism management education. Tourism learning is inherently embedded in industrial practice, social interaction, and complex decision-making processes, where students are frequently required to engage in project-based activities, community collaboration, service-learning, and industry partnerships. In such contexts, learners are expected to deal with open-ended tasks, dynamic situations, and multi-actor coordination (Chen et al., 2022; Croft and Wang, 2025; Nimri et al., 2024). From this perspective, learning in tourism management is not merely a process of knowledge acquisition, but a continuous process in which students coordinate actions, reflect on experiences, and develop professional judgment within authentic or quasi-authentic environments (Liang, 2021; Villarroel Henríquez et al., 2025). Accordingly, experiential learning should not be regarded as a supplementary pedagogical approach, but rather as one of the core modes of learning in tourism management education (Doğantan, 2023; Sundman et al., 2025).

However, the notion of “sustainability” emphasized in ESD does not imply that competence development can be achieved through a single or short-term experiential activity. Instead, it highlights whether students are able to gradually develop transferable and extensible professional capacities through sustained engagement over time (Olsson et al., 2022; Sundman et al., 2025). Therefore, rather than focusing solely on whether experiential activities occur, it is more critical to examine how students maintain participation, respond to challenges, and progressively transform episodic experiences into long-term developmental resources within supportive environments (Li and Fan, 2026). In this sense, sustainable experiential learning in tourism management should be understood not as isolated instructional events, but as a learning ecosystem that depends on continuous interaction and support conditions (Annelin and Boström, 2024; Höffken and Lazendic-Galloway, 2024; Sundman et al., 2025).

### 2.2 Dual support: teacher instructional guidance and student participation support

While experiential learning provides students with authentic and complex practice settings, whether such learning can be sustained largely depends on the presence of stable and effective support mechanisms throughout the learning process. Accordingly, this study conceptualizes support as comprising two interrelated dimensions: teacher instructional guidance and student participation support.

(1) Teacher instructional guidance represents a crucial external condition for the orderly progression of experiential learning. In tourism management contexts, the role of teachers extends beyond task assignment or outcome evaluation. Through task decomposition, process scaffolding, feedback regulation, and reflective guidance, teachers help students construct executable action pathways in complex situations (Allagui, 2024; Liu et al., 2023). On the one hand, clear task structures, staged goals, and process-oriented feedback can reduce confusion in the early stages of learning and enhance students’ perceived comprehensibility and controllability of tasks. On the other hand, when students encounter failure, setbacks, or uncertainty during practice, teachers can help reinterpret errors, regulate contextual risks, and clarify the meaning of learning experiences. Such guidance enables students to reframe pressure from a perceived “threat of incompetence” into a “manageable learning challenge” (Narciss and Alemdag, 2025). Therefore, teacher instructional guidance not only serves a task-supporting function but also plays an essential role in psychological regulation, particularly in maintaining students’ willingness to participate under uncertainty (Senden et al., 2023).
(2) ()Student participation support is primarily manifested through peer interaction and collaborative participation. Experiential learning in tourism management often requires students to jointly complete tasks such as information collection, on-site communication, task coordination, and solution design. As a result, the learning process is inherently dependent on peer collaboration (Parmar et al., 2025). Student participation support is reflected not only in the sharing of knowledge, experiences, and strategies, but also in emotional responses, responsibility sharing, and mutual encouragement when facing pressure. Through such collaborative participation, students are able to reduce feelings of isolation in complex tasks and develop a social foundation for sustained action (Kim, 2024). In other words, within experiential learning in tourism management, teacher instructional guidance primarily represents structured and organizational support within the instructional system, whereas student participation support represents interactive and relational support within the learning community. Importantly, these two forms of support are not independent. Rather, they operate simultaneously within the same learning context and jointly constitute the core support system of a sustainable experiential learning ecosystem (Holst et al., 2024; Li, 2025).

### 2.3 Internalization of support: from psychological safety to sustained engagement

External support alone is insufficient to automatically generate sustainable learning outcomes. More critically, it depends on how teacher instructional guidance and student participation support are subjectively perceived, interpreted, and further internalized by students as psychological resources for sustained engagement. Based on this perspective, the present study conceptualizes psychological safety, self-efficacy, and participation interest as key entry points for understanding the process of support internalization.

(1) ()Psychological safety serves as a foundational condition for students to engage in deeper experiential learning. In authentic or quasi-authentic tourism management contexts, students are often required to express ideas publicly, assume role responsibilities, and respond to uncertain feedback. As a result, they may experience concerns about making mistakes, being negatively evaluated, or lacking competence (Narciss and Alemdag, 2025). When teachers reduce the perceived threat of failure through supportive feedback, and peers alleviate individual pressure through shared responsibility, students are more likely to develop a psychological state characterized by “being able to try, make mistakes, and continue participating.” This form of psychological safety should not be understood as a background atmosphere, but rather as a critical precondition that shapes whether students are willing to further engage in practice, assume responsibilities, and sustain participation (Senden et al., 2023).
(2) Self-efficacy and participation interest further constitute the internal driving forces of sustained engagement. Self-efficacy refers to students’ judgments of their capability to accomplish tasks. In experiential learning contexts, when students are able to complete staged tasks with support and receive positive responses from teachers and peers, they are more likely to develop a sense of “I am capable,” thereby strengthening their self-efficacy beliefs (Liu et al., 2023). Once established, such beliefs can enhance students’ willingness to invest effort and persist when encountering new challenges (Shum and Fryer, 2026). At the same time, participation interest should not be understood as a temporary preference, but as a form of learning motivation that is gradually activated and maintained through meaningful participation experiences (Guo and Fryer, 2025). When students perceive that they can understand tasks, cope with challenges, and recognize the developmental value of their experiences, their participation interest is more likely to be strengthened. Moreover, existing research suggests that self-efficacy and interest function in a mutually reinforcing manner, forming a cyclical process that supports sustained engagement over time (Lee et al., 2024). Accordingly, psychological safety, self-efficacy, and participation interest together constitute a crucial bridge through which support mechanisms are transformed into sustained engagement.
(3) When students consistently experience that they are able to try, capable of succeeding, engaged in meaningful activities, and supported by others in experiential learning, they are more likely to develop relatively stable positive psychological experiences, including a sense of meaning, belonging, and controllability (Klainin-Yobas et al., 2021; Zhu et al., 2022). Such psychological states not only help maintain current learning engagement, but also encourage students to regard experiential learning as an important pathway for their professional development. From a process-oriented perspective, therefore, sustainable experiential learning should not be understood as being driven solely by external instructional arrangements. Rather, it emerges as a sustained engagement process that is gradually internalized through the joint functioning of teacher instructional guidance and student participation support, mediated by psychological mechanisms such as psychological safety, self-efficacy, and participation interest. In the present study, these psychological factors are not treated as parallel variables, but as components of an interconnected process-oriented mechanism, in which psychological safety provides the foundational condition for participation, while self-efficacy and participation interest jointly sustain ongoing engagement.

### 2.4 Analytical perspective of the present study

Based on the above discussion, this study conceptualizes sustainable experiential learning in tourism management education as a learning ecosystem co-constructed through multi-actor interaction. Within this ecosystem, teacher instructional guidance and student participation support constitute the external support foundation, while psychological safety, self-efficacy, and participation interest represent the key processes through which support is subjectively interpreted and internalized by students. Ultimately, this process is associated with whether students are able to maintain sustained engagement, develop positive psychological experiences, and gradually promote the development of professional competence. Although existing research has examined experiential learning, teacher support, peer collaboration, and related psychological mechanisms separately, there remains a lack of process-oriented explanation of how these factors are interconnected within the same experiential learning context in tourism management education and how they jointly shape a sustainable experiential learning ecosystem (Annelin and Boström, 2024; Schutte et al., 2025).

Building on the above discussion, this study develops a process-oriented analytical perspective, conceptualizing experiential learning in tourism management as a dynamic learning ecosystem structured by the pathway of “support–internalization–sustained engagement.” Within this framework, teacher instructional guidance and student participation support function as external contextual conditions, whereas psychological safety, self-efficacy, and participation interest represent the key mechanisms through which support is interpreted and transformed by learners. This analytical perspective provides a theoretical reference for the subsequent qualitative analysis, enabling an explanation of how support is constructed, internalized, and translated into sustained engagement within specific learning contexts.

## 3 Materials and methods

### 3.1 Research design

To gain an in-depth understanding of how teacher instructional guidance and student participation support are enacted within experiential learning contexts in tourism management education, and how these forms of support are interpreted and transformed into psychological resources for sustained engagement, this study adopts a qualitative research design. The analysis is grounded in the methodological principles of constructivist grounded theory, emphasizing the exploration of participants’ lived experiences and the examination of interactional mechanisms and processes of meaning construction within learning contexts (Charmaz and Thornberg, 2021; Charmaz, 2014). Building on the theoretical framework outlined above, this study adopts a “support–internalization–sustained engagement” analytical perspective and conceptualizes sustainable experiential learning as a process. Specifically: (1) teacher instructional guidance and student participation support constitute the external support mechanisms; (2) these forms of support are gradually internalized through learners’ subjective interpretations into psychological resources such as psychological safety, self-efficacy, and participation interest, which are further associated with students’ sustained engagement and learning experiences. Accordingly, this study does not aim to test predefined relationships among variables. Instead, it employs qualitative analysis to examine how support mechanisms are constructed within specific learning contexts, how they are internalized by learners, and how they are associated with sustained engagement as a dynamic and process-oriented phenomenon.

The theoretical constructs presented in section “2 Theoretical framework” are treated as sensitizing concepts rather than predefined categories. They served to enhance theoretical sensitivity during analysis without constraining category emergence from data.

### 3.2 Participants

To comprehensively understand learning processes under varying support experiences and levels of engagement, this study employed purposive sampling to recruit participants with substantial experience in experiential learning (Patton, 2015). The sample was drawn from two comprehensive universities in Jeollabuk-do, South Korea, and included a total of 10 tourism management teachers and 10 undergraduate students. To ensure diversity within the sample, teacher participants were categorized according to teaching experience into three stages: early-career (4–5 years, *n* = 3), mid-level (6–7 years, *n* = 3), and senior (8–10 years, *n* = 4). This classification enabled the study to capture variations in teacher instructional guidance practices across different stages of professional development. Student participants were selected through preliminary communication to ensure variation in their perceived teacher instructional guidance, experiences of student participation support, and levels of engagement, including participation interest, self-efficacy, and sustained engagement. This procedure enhanced the heterogeneity of the sample in terms of support experiences and engagement trajectories. Such a sampling strategy facilitates the examination of learning processes across different support conditions and participation states, thereby providing a more comprehensive understanding of the relationships among support mechanisms, psychological internalization processes, and sustained engagement. Basic demographic characteristics of the participants are presented in Table 1.

**TABLE 1:** Demographic profile of teacher and student participants.

Participant group	*N*	Key characteristics of participants
Teachers	10	Teaching experience ranged from 4 to 10 years, covering early-career (4–5 years), mid-level (6–7 years), and senior (8–10 years) stages to ensure variation in instructional practices. Participants were from tourism-related disciplines (e.g., tourism management, hospitality management, cultural heritage tourism, tourism marketing) at two comprehensive universities in Jeollabuk-do, South Korea. All had substantial experience in experiential teaching (e.g., fieldwork, service-learning, industry collaboration projects).
Students	10	Undergraduate students (Years 1–4) majoring in tourism management or related disciplines. All participants had completed at least one in-depth experiential learning course (e.g., internships, community-based tourism projects). Purposeful sampling ensured variation in perceived teacher guidance, peer support, and levels of engagement, self-efficacy, and learning interest.

### 3.3 Data collection

This study employed semi-structured interviews as the primary method of data collection. Semi-structured interviews are particularly suitable for capturing participants’ experiences, emotions, and processes of meaning construction in complex learning contexts, especially for examining support mechanisms and psychological processes in experiential learning (Creswell and Poth, 2018). The interview protocol was designed based on the analytical framework of this study, focusing on the following aspects: (1) teachers’ instructional practices, decision-making logic, and support strategies in experiential teaching; (2) students’ participation experiences in project-based learning, field practice, and collaborative tasks, as well as their perceived student participation support; (3) the relationships between support mechanisms and psychological processes, including the formation and development of psychological safety, self-efficacy, and participation interest; and (4) participants’ understandings of a sustainable experiential learning environment. The interviews were conducted using a progressive questioning approach, with follow-up probes employed to distinguish participants’ descriptions of support practices, emotional experiences, and learning behaviors. Responses involving multiple dimensions were subsequently separated into distinct meaning units during analysis.

Each interview lasted approximately 45 min and was audio-recorded with participants’ consent and transcribed verbatim. All interviews were conducted in Korean and transcribed in the original language. Coding and analysis were based on the original transcripts. The research team translated selected quotations into English and cross-checked them to ensure semantic consistency. All data were managed and analyzed using NVivo 14. To ensure participant confidentiality, teachers and students were anonymized using the identifiers “T1–T10” and “S1–S10,” respectively. Examples of the interview protocol and sampling framework are provided in Supplementary Tables 1, 2, while detailed coding procedures and analytical processes are presented in Supplementary Table 3.

### 3.4 Data analysis and trustworthiness

Data analysis followed the procedures of constructivist grounded theory, employing a three-stage coding process consisting of open coding, axial coding, and selective coding (Charmaz and Thornberg, 2021).

(1) ()Open coding: Interview transcripts were analyzed line-by-line to identify initial concepts related to teacher instructional guidance, student participation support, and associated psychological experiences, including psychological safety, self-efficacy, and participation interest. A total of 92 initial concepts were generated.
(2) ()Axial coding: Through constant comparative analysis, the initial concepts were systematically integrated and categorized. This stage focused on examining the relationships between different types of support and psychological mechanisms, as well as their roles in sustained engagement, resulting in the development of 16 categories.
(3) Selective coding: The core category of “sustainable experiential learning ecosystem” was identified to integrate teacher instructional guidance, student participation support, and their internalization processes. Based on this, an overall explanatory framework was constructed to illustrate how support mechanisms are transformed into sustained engagement through psychological processes. Detailed coding procedures and illustrative examples are provided in Supplementary Table 3.

To ensure the trustworthiness and rigor of the analysis, several strategies were employed (Strauss and Corbin, 1998): (1) Inter-coder agreement: Two researchers independently coded approximately 30% of the transcripts. Discrepancies were resolved through iterative discussion to enhance analytical consistency and stability; (2) Member checking: Preliminary findings were shared with selected participants to verify the credibility of interpretations; (3) Theoretical saturation: No new conceptual categories emerged during axial coding after the 9th teacher interview and the 8th student interview. Saturation was evaluated at the category level, confirming stability across all 16 categories in terms of properties and dimensions; and (4) Reflexive memoing: Reflexive analytic memos were maintained throughout the research process to enhance transparency and support continuous reflection. Given that one author is affiliated with one of the participating institutions, reflexive strategies including peer debriefing, iterative coding comparison, and audit trail documentation were employed to mitigate potential insider bias and power asymmetry. Due to the length and structural constraints of the manuscript, detailed coding examples and Supplementary Table 1–6 are not fully reproduced in the main text but are available in the Supplementary materials.

## 4 Findings

Based on the three-stage coding process of constructivist grounded theory, a total of 92 initial concepts were identified during the open coding stage. These concepts were derived from detailed accounts provided by teachers and students regarding specific practices, interactional experiences, and related psychological processes in experiential learning contexts. During axial coding, through constant comparative analysis, these concepts were further integrated into 16 categories, revealing the internal relationships among teacher instructional guidance, student participation support, and associated psychological mechanisms. In the selective coding stage, “sustainable experiential learning ecosystem” was identified as the core category, integrating different forms of support and their internalization processes into an overall explanatory framework. On this basis, three interrelated core themes were identified: (1) the dual-pathway structure of teacher instructional guidance; (2) the interactive coordination mechanism of student participation support; and (3) the internalization of support and the process of sustained engagement. The thematic structure of these findings is summarized in Table 2. To further clarify the dual-perspective structure, teacher and student experiences are systematically compared across key dimensions of support mechanisms and internalization processes. Table 3 synthesizes cross-case patterns derived from the coding results (Supplementary Tables 4–6) and presents a conceptual comparison between teacher and student perspectives.

**TABLE 2:** Overview of the thematic structure of the sustainable experiential learning ecosystem.

Core theme	Subcategory	Conceptual meaning
Dual-pathway structure of teacher instructional guidance	Structured guidance	Teachers guide students through task design, role allocation, key process checkpoints, and explicit evaluation criteria, enabling students to gradually develop a sense of competence and clear action pathways within contexts of controllable challenge.
	Reflective–emotional guidance	Through feedback-oriented dialogue, risk regulation, meaning-making, and emotional scaffolding, teachers help students reduce uncertainty-related pressure and sustain the psychological momentum required for continued participation.
Interactive coordination mechanism of student participation support	Cognitive coordination	Through peer interaction, students enhance problem-solving efficiency and participation quality by sharing information, complementing strategies, coordinating roles, and collectively reflecting on learning situations.
	Emotion-regulatory support	Peers stabilize emotional labor during learning through emotional reassurance, empathic responses, buffering of frustration, and shared responsibility, thereby preventing disengagement from participation.
Internalization pathways of support and sustainable participation cycle	Formation of psychological safety	The combined influence of teacher instructional guidance and student participation support reduces evaluation anxiety and contextual risk, enabling students to take learning-related risks and assume responsibility for their learning.
	Interest–self-efficacy reinforcement	Experiential success and recognized learning efforts strengthen students’ beliefs in their competence, which in turn activate deeper and more sustained participation interest.
	Psychological wellbeing and sustained engagement	When a sense of meaning, belonging, and perceived control is continuously maintained, students gradually develop stable psychological wellbeing and long-term willingness to engage.

**TABLE 3:** Comparative analysis of teacher and student perspectives.

Dimension	Teacher perspective	Student perspective
Structured support	Designs task scaffolding, staging, and evaluation criteria	Experiences clearer task pathways and reduced uncertainty
Emotional/reflective support	Reframes failure and guides meaning-making	Experiences reduced anxiety and normalized mistakes
Cognitive coordination	Organizes collaborative structure and task distribution	Engages in peer learning, imitation, and strategy sharing
Emotion regulation	Provides indirect emotional scaffolding through pedagogy	Experiences shared responsibility and emotional buffering
Internalization role	Creates enabling conditions for engagement	Experiences psychological safety → self-efficacy → engagement cycle

### 4.1 Dual pathways of teacher guidance

In experiential learning contexts within tourism management education, teachers do not merely assume the role of instructional organizers. Rather, they provide continuous structural and contextual support within complex learning environments, enabling students to sustain action and learning progression under conditions of uncertainty. The findings indicate that teacher instructional guidance operates through two complementary pathways: (1) structured guidance oriented toward task progression; and (2) reflective–emotional guidance centered on regulating uncertain situations. These two pathways correspond to the dimensions of capability development and psychological regulation, respectively, and together constitute essential external mechanisms that support students’ sustained engagement.

#### 4.1.1 Structured guidance and capability development

Interview data indicate that teachers generally perceive the effectiveness of experiential learning not in terms of whether authentic contexts are provided, but in whether students are able to develop clear and executable action pathways within complex situations. Accordingly, in practice, teachers transform originally open-ended and uncertain learning tasks into structured learning processes through task decomposition, staged progression, role allocation, and the clarification of evaluation criteria.

As described by T5 in a destination-based service-learning project: “We break the whole project into several stages, such as entering the context, problem identification, interviews, data organization, and final presentation. Each stage has clear requirements.” Such structured arrangements enable students to gradually develop a sense of “what to do next” within complex environments, thereby significantly reducing initial uncertainty and cognitive load. Therefore, this form of guidance not only shapes task execution but also plays an important role in the development of students’ sense of capability. S8 described that during the early stages of an industry collaboration project, incomplete information and complex communication led to a strong sense of uncertainty and anxiety: “At the beginning, I really didn’t know what to do or even what to ask.” However, after the teacher provided interview templates, task allocation frameworks, and staged guidance, the student gradually established a clearer action pathway: “By following the steps given by the teacher, I gradually understood what I was doing.” Similarly, S4 noted: “Once there are clear steps, you don’t feel as overwhelmed, and it feels like something that can be completed step by step.” Cross-case analysis suggests that structured guidance operates through a process of “reducing complexity – providing pathways – accumulating experience,” through which students gradually build successful experiences in practice. These experiences not only improve task performance but also strengthen students’ beliefs in their own capabilities. Therefore, structured guidance is not only a form of task-level support but also a critical precondition for the development of self-efficacy.

#### 4.1.2 Reflective–emotional guidance under uncertainty

Complementing structured guidance is teachers’ provision of reflective and emotional support in uncertain situations. During experiential learning, students frequently encounter challenges such as unsuccessful interviews, rejected proposals, and deviations in field execution. These situations are often accompanied by strong emotional fluctuations and may even lead to disengagement. The findings indicate that, in such situations, teachers typically do not provide direct answers. Instead, they guide students through reflective dialogue.

As T7 explained: “When problems occur, I don’t tell them the answer directly. I ask them what happened and whether there are other possible approaches.” This approach helps students move from single-cause attribution to multi-dimensional understanding, thereby preventing them from attributing failure solely to a lack of ability. S3 described a team failure experience: “At that time, I felt that I hadn’t done well, but the teacher helped us analyze what was due to the situation and what could be adjusted.”

In addition, teachers regulate students’ emotional experiences by emphasizing the meaning of the learning process. T9 stated: “Experiential learning is not about doing things perfectly, but about growing through the process.” S6 also noted: “The teacher said this is part of learning, so I became more willing to try again.” Thus, reflective–emotional guidance operates through a process of “reinterpreting failure – reducing perceived threat – constructing meaning,” enabling students to sustain engagement in uncertain situations. This form of guidance not only alleviates emotional pressure but also helps students develop a developmental learning perspective, thereby strengthening their willingness to remain engaged over time. Additional representative quotations for this theme are presented in Supplementary Table 4.

### 4.2 Interactive mechanisms of student support

Compared with the structural and contextual guidance provided by teachers, student participation support is primarily manifested as interactional processes within the learning community. The findings indicate that, in experiential learning, student support operates mainly through two interrelated mechanisms: cognitive coordination and emotion-regulatory support. These mechanisms are intertwined within collaborative processes and jointly sustain the continuity of learning progression.

#### 4.2.1 Cognitive coordination in collaboration

During project-based experiential learning, students typically complete complex tasks through collaborative division of labor. S2 noted: “We divide the work based on what each person is good at—some organize data, some handle communication, and then we discuss together.” Such division of roles not only improves task efficiency but also provides a foundation for information integration and strategy formation.

S7 stated: “By seeing how others communicate with clients, I learn some methods, and I feel more confident the next time.” S1 added: “Sometimes others have better approaches, and I directly apply them to my own part.” Further analysis indicates that cognitive coordination is not limited to task-level collaboration, but also involves the sharing and transformation of experience.

Through observation, imitation, and discussion, students transform individual experiences into collective knowledge, which is subsequently internalized as personal capability. Therefore, cognitive coordination enables experiential learning to shift from an individual activity to a collective learning process, in which shared strategies and accumulated experiences support students’ ongoing participation.

#### 4.2.2 Emotion-regulatory support through shared responsibility

In high-intensity experiential learning contexts, students face not only task-related pressure but also substantial emotional demands. The findings indicate that emotional support among peers plays a critical role in sustaining engagement. S5 described: “Sometimes it’s really exhausting, but when we do it together, it feels like we can keep going.” This experience of “shared responsibility” allows students to reinterpret pressure as a collective situation rather than an individual burden, thereby reducing psychological strain. S8 noted: “They first point out what we did well, and then we think together about how to improve.” S6 added: “When someone discusses it with you, you don’t feel as anxious.” Thus, emotion-regulatory support stabilizes students’ emotional states during learning and provides a social foundation for continued action. In essence, this mechanism reduces the cost of participation, enabling students to maintain engagement even under pressure. Further illustrative evidence is provided in Supplementary Table 5.

### 4.3 Internalization of support and sustained engagement

During the selective coding stage, the findings further indicate that teacher instructional guidance and student participation support do not remain at the level of external task-related support. In this study, sustained engagement refers to continuous, voluntary, and active participation in experiential learning activities over time, extending beyond task completion to include proactive involvement and repeated engagement. Rather, through continuous interaction and accumulated experience, these forms of support are gradually interpreted and internalized by students as psychological resources. Participants’ accounts suggest that this transformation is not a one-time occurrence, but a dynamic process formed through repeated cycles of participation, feedback, and adjustment.

Specifically, this process demonstrates a clear stage-based structure: (1) under the combined influence of teacher and peer support, students gradually develop psychological safety, thereby lowering the threshold for participation. Some participants initially reported hesitation and uncertainty during early stages of participation, particularly when tasks were unfamiliar or ambiguous, before gradually developing psychological safety through interactional support; (2) through continued participation, students accumulate staged success experiences, which strengthen self-efficacy and activate participation interest; and (3) through stable participation, students develop relatively enduring positive psychological experiences, which in turn further support subsequent engagement. Accordingly, psychological safety, self-efficacy, participation interest, and psychological experiences should not be treated as independent variables, but rather as components of an interconnected and mutually reinforcing process-oriented mechanism.

#### 4.3.1 Psychological safety as a starting condition

Students consistently reported that, in experiential learning, the primary constraint on participation is not task difficulty per se, but concerns about failure consequences, evaluation by others, and uncertainty in the learning context. In the absence of sufficient support, such concerns often lead to avoidance behaviors or reduced engagement.

S3 stated: “Only when I feel that making mistakes is allowed do I dare to try new things.” S7 similarly noted: “If the team atmosphere is safe, I don’t worry too much about making mistakes.” These findings indicate that psychological safety is not derived from a single source, but is jointly constructed through teacher instructional guidance and student participation support. On the one hand, teachers reduce the perceived threat of failure through non-evaluative feedback, emphasizing the importance of the learning process, and reframing errors. On the other hand, peers reduce individual pressure through shared responsibility and emotional support. S6 explained: “If everyone shares the responsibility, it doesn’t feel like I alone am responsible for the outcome.” S1 added: “When everyone is trying, making mistakes doesn’t feel like a serious issue.” Through such supportive interactions, students gradually reinterpret uncertainty from a “risk to be avoided” into a “manageable learning situation,” thereby becoming more willing to express ideas, assume roles, and engage in complex tasks. Therefore, psychological safety should not be understood as an abstract background climate, but as an experiential outcome constructed through concrete interaction. It lowers the psychological threshold for entering experiential learning and provides the necessary precondition for the subsequent development of self-efficacy and participation interest, serving as the starting point of support internalization.

#### 4.3.2 Reinforcing self-efficacy and participation interest

Following the formation of psychological safety, students are more likely to enter a state of sustained engagement and gradually accumulate successful experiences through active participation. The findings indicate that these experiences form a cyclical structure of: engagement → success → enhanced confidence → renewed engagement.

S8 stated: “After completing an important task, I felt that I could actually do it, and then I wanted to continue participating.” This suggests that self-efficacy is not pre-existing but is gradually developed through concrete practice and accumulated success experiences. External feedback from teachers and peers plays a critical role in this process. S2 noted: “When others recognize your contribution, you feel that you really play a role in the team.” S4 added: “When the teacher says you did this part well, it gives you more confidence to continue.” Such positive feedback enables students to clearly perceive improvements in their capabilities, thereby strengthening their belief in their own competence. On this basis, participation interest is further activated. Students gradually shift from externally driven task completion to intrinsic engagement in the learning process itself. S7 stated: “At first, I was just completing tasks, but later I found the process itself quite interesting.” S5 added: “Once I started to understand the methods, I wanted to try different approaches.” Thus, self-efficacy and participation interest exhibit a mutually reinforcing relationship: success experiences enhance capability beliefs, which in turn promote deeper engagement, forming an internal motivational mechanism that sustains participation over time. This cyclical structure enables students to maintain action in changing contexts and gradually strengthen their agency in learning.

#### 4.3.3 Psychological experiences and sustained engagement

When psychological safety and the self-efficacy–participation interest cycle are stably maintained, students gradually develop relatively enduring positive psychological experiences. These experiences are not limited to short-term emotional responses, but reflect an integrated perception of meaning, social relationships, and control in learning.

S9 stated: “Although the process is tiring, it feels meaningful, and I look forward to the next experience.” S5 noted: “When the project progresses smoothly, I feel a strong sense of achievement and want to continue similar activities.” These psychological experiences can be understood in three dimensions: (1) sense of meaning: linking learning activities to professional development; (2) sense of belonging: feeling accepted and supported within the group; (3) sense of controllability: perceiving the ability to manage tasks and outcomes. S6 stated: “When the team works well together, it feels like I am learning in a very good environment.” S8 added: “When I know I can handle things, I don’t feel as anxious.” Importantly, in this study, psychological experiences are not merely outcomes of learning but function as psychological resources that further sustain subsequent engagement. When students continuously experience meaning, belonging, and controllability, their participation becomes more stable, and they are more likely to actively seek new experiential opportunities. From a process-oriented perspective, therefore, sustained engagement is not maintained by external requirements alone. Rather, it emerges as a dynamic process in which support mechanisms are gradually internalized into psychological resources, enabling learners to actively sustain their own engagement. This process further substantiates the overall mechanism of “support–internalization–sustained engagement.” Supporting evidence for the internalization process is presented in Supplementary Table 6.

**FIGURE 1:**
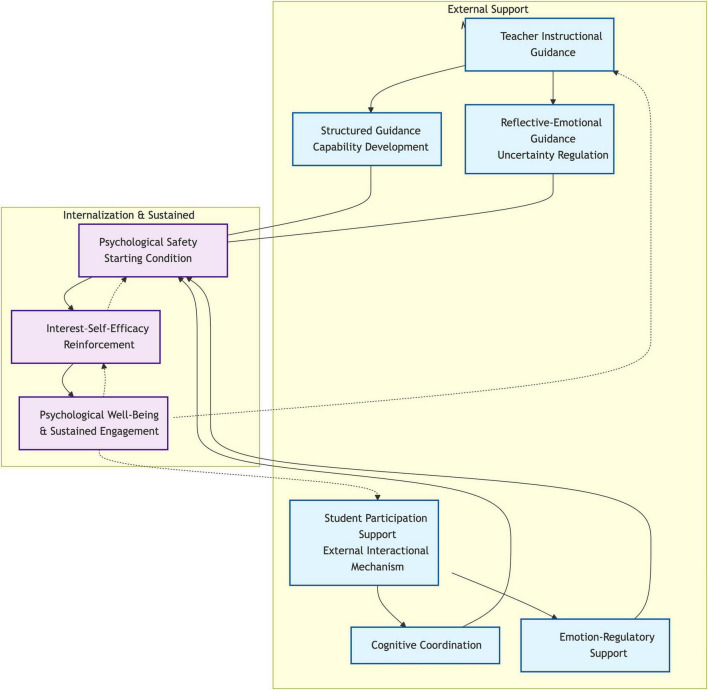
Sustainable experiential learning ecosystem model. Student participation support is conceptualized exclusively as an external interactional mechanism. Solid arrows indicate primary process pathways, while dashed arrows represent recursive and reinforcing relationships. The model reflects a process-oriented mechanism rather than a linear causal structure.

Based on the integrated findings, a process-oriented model of the sustainable experiential learning ecosystem is proposed (see Figure 1).

## 5 Discussion

### 5.1 Teacher guidance as external support for sustained engagement

Teacher instructional guidance in experiential learning contexts within tourism management education operates through two interrelated pathways—structured guidance and reflective–emotional guidance—which function continuously throughout the learning process. These pathways simultaneously provide organizational structure and psychological regulation in complex and uncertain learning environments, forming a critical external foundation for sustained engagement (Chen et al., 2024).

(1) ()Structured guidance transforms originally open-ended and uncertain learning tasks into processes with clear and executable pathways through task decomposition, staged progression, role allocation, and explicit evaluation criteria. As demonstrated in the findings, this process operates through “reducing complexity – providing pathways – accumulating experience,” enabling students to gradually develop understanding and control over tasks. Through continuous practice, students build a foundation of capability, which aligns with prior research emphasizing the importance of structured support in facilitating competence development in experiential learning contexts (Chen et al., 2024; Rashed et al., 2025). Importantly, such structured support not only facilitates task progression but also provides experiential resources for the subsequent development of self-efficacy, which has been widely identified as a key factor influencing sustained learning engagement (Annelin and Boström, 2024; Shengyao et al., 2024).
(2) Reflective–emotional guidance plays a critical regulatory role under conditions of uncertainty. When students encounter deviations or setbacks in practice, teachers guide them through reflective dialogue and the reinterpretation of failure. This process enables students to move from single-dimensional ability attribution toward a more multidimensional understanding of situational and process-related factors, thereby reducing the perceived threat of failure. Furthermore, by emphasizing the meaning of the learning process itself, teachers help sustain students’ willingness to participate. This mechanism corresponds closely with the process identified in the findings—“reinterpreting failure – reducing perceived threat – constructing meaning”—and is consistent with existing research highlighting the role of feedback and error-based learning in supporting adaptive learning processes (Narciss and Alemdag, 2025). Taken together, the dual-pathway structure of teacher instructional guidance operates at both the action level and the psychological level. Through their combined and coordinated effects, these two forms of guidance provide stable support for students’ sustained engagement in complex experiential learning contexts. In this sense, teacher instructional guidance constitutes a key external condition within the sustainable experiential learning ecosystem, reinforcing the importance of structured and emotionally responsive teaching practices in ESD-oriented learning environments (Holst et al., 2024; Shen et al., 2024).

### 5.2 Student support and learning process maintenance

Compared with the structural and contextual guidance provided by teachers, student participation support is more prominently manifested as interactional processes within the learning community. The findings of this study indicate that, in experiential learning, student support operates primarily through two mechanisms—cognitive coordination and emotion-regulatory support—which jointly sustain the continuity of learning progression during collaboration.

(1) ()Cognitive coordination transforms learning from an individual activity into a collaboratively constructed process. Through division of labor, experience sharing, and strategy discussion, students are able to integrate information, optimize action pathways, and convert others’ experiences into their own actionable resources. As demonstrated in the findings, this process not only improves task efficiency but also provides an important foundation for the development of students’ sense of capability in practice. This interpretation aligns with prior research emphasizing the role of collaborative learning and peer interaction in facilitating engagement and competence development (Chen et al., 2024; Li, 2025; Parmar et al., 2025).
(2) ()Emotion-regulatory support reduces the psychological burden associated with participation in high-intensity and uncertain learning contexts. Through mutual encouragement, shared responsibility, and emotional responsiveness, students transform individualized pressure into a collectively managed situation, thereby stabilizing the participation process. This experience of “shared responsibility” helps alleviate anxiety and uncertainty, enabling students to sustain action through ongoing interaction. Such findings are consistent with research highlighting the importance of peer-based emotional support in maintaining engagement and wellbeing in collaborative learning environments (Kim, 2024; Rashed et al., 2025).

Importantly, cognitive coordination and emotion-regulatory support do not operate independently, but are intertwined within collaborative processes. Cognitive coordination facilitates task progression at the action level, while emotion-regulatory support stabilizes participation at the psychological level. Through their combined effects, student participation support not only constitutes the interactional foundation for learning progression but also provides essential psychological support for sustained engagement.

### 5.3 Internalization of support and the mechanism of sustained engagement

Under the combined influence of teacher instructional guidance and student participation support, the present study further illustrates how support is gradually internalized into psychological resources that sustain engagement over time. The findings indicate that this internalization process follows a progressive structure, moving from psychological safety, to self-efficacy and participation interest, and ultimately to relatively stable psychological experiences. First, psychological safety serves as the starting condition for students’ deeper engagement. Through teachers’ non-evaluative feedback and peers’ shared responsibility, students gradually develop a psychological state in which “trying and making mistakes is acceptable,” thereby lowering the threshold for participation. As indicated in the findings, such psychological safety is not a pre-existing environmental condition, but is progressively constructed through concrete interaction. This interpretation is consistent with prior research emphasizing the role of supportive environments in enabling risk-taking and participation in learning processes (Senden et al., 2023). Building on this foundation, self-efficacy and participation interest are gradually developed and mutually reinforced through sustained engagement. Through the completion of staged tasks and the positive feedback provided by teachers and peers, students accumulate experiences of “being able to accomplish tasks,” which strengthens their beliefs in their own capabilities (Chen et al., 2024). At the same time, as students deepen their understanding of tasks and perceive improvements in their abilities, their participation interest is activated and maintained (Guo et al., 2023). These two factors form a cyclical relationship in practice, enabling students to sustain engagement in dynamically changing contexts. This finding aligns with existing research highlighting the reciprocal relationship between self-efficacy and interest in learning processes (Lee et al., 2024; Rashed et al., 2025; Shen et al., 2024; Shum and Fryer, 2026).

As this process becomes more stable, students gradually develop relatively enduring positive psychological experiences, including a sense of meaning, sense of belonging, and sense of controllability. Importantly, rather than functioning merely as outcomes, these psychological experiences are further transformed into psychological resources that support subsequent participation. When students continuously experience meaning, belonging, and control, they become more inclined to sustain engagement and to actively seek new experiential learning opportunities. This perspective is consistent with research suggesting that psychological wellbeing plays an active role in maintaining engagement and adaptive functioning in learning contexts (Klainin-Yobas et al., 2021; Zhu et al., 2022).

From a process-oriented perspective, sustained engagement emerges as a dynamic process in which support is gradually internalized into psychological resources, enabling learners to actively sustain their own participation over time (Shen et al., 2024), forming a “support–internalization–sustained engagement” pathway within experiential learning in tourism management education. It should also be acknowledged that this internalization process may be additionally shaped by institutional learning culture and participants’ retrospective interpretation of their learning experiences.

### 5.4 Process mechanism of the sustainable experiential learning ecosystem

Based on the above analysis, this study presents a process-oriented explanation of the formation of the sustainable experiential learning ecosystem in tourism management education. The findings indicate that sustainable experiential learning is not determined by isolated instructional arrangements or short-term participation. Rather, it is gradually developed through multi-actor interaction and the accumulation of learning experiences over time. Within this process, teacher instructional guidance and student participation support constitute the foundational forms of external support. Through the combined effects of structural guidance and interactional support, these mechanisms provide stable conditions for the progression of learning. On this basis, such support is progressively interpreted and internalized by learners through experience accumulation, transforming into psychological resources such as psychological safety, self-efficacy, and participation interest, and further contributing to the formation of relatively stable positive psychological experiences. Importantly, these psychological resources do not function merely as outcomes, but in turn sustain and reinforce students’ sustained engagement, enabling the learning process to exhibit a cyclical and developmental pattern. Therefore, sustainable experiential learning can be understood as a dynamic process constructed through the interaction between external support and internal psychological mechanisms. This process is also consistent with key ESD 4.7 competencies, particularly systems thinking (interaction among multiple actors in learning ecosystems), anticipatory competence (engagement under uncertainty and evolving experiential contexts), and normative competence (reflection on learning meaning, responsibility, and value formation). Within this process, support does not operate as a one-time input, but is continuously constructed and reinforced through ongoing interaction and accumulated experience, thereby enabling learning participation to be maintained over the long term.

### 5.5 Theoretical contributions

Based on the above findings, the theoretical contributions of this study can be summarized in three aspects; (1) By adopting a dual-perspective approach that incorporates both teachers’ and students’ experiences, this study shifts the understanding of experiential learning in tourism management from an outcome-oriented perspective to a process-oriented analysis. It reveals how support mechanisms are gradually transformed into sustained engagement through interaction and the accumulation of experience in specific learning contexts. This perspective contributes to a deeper understanding of sustainable experiential learning; (2) This study proposes and specifies the dual-pathway structure of teacher instructional guidance, consisting of structured guidance and reflective–emotional guidance, and demonstrates their coordinated roles at both the action-support and psychological-regulation levels. This finding extends current understandings of the role of teachers in experiential learning contexts; and (3) This study provides a process-oriented explanation of the mechanisms underlying student participation support. The findings indicate that cognitive coordination and emotion-regulatory support not only facilitate task progression, but also influence sustained engagement through process-based mechanisms such as psychological safety, self-efficacy, and participation interest. In this way, the study offers a more systematic explanation of the role of the learning community in experiential learning.

### 5.6 Limitations and future research

Despite advancing the understanding of the relationship between support mechanisms and sustained engagement in experiential learning within tourism management education, this study has several limitations; (1) The study adopts a cross-sectional qualitative design, which limits its ability to directly capture the dynamic evolution of psychological safety, self-efficacy, and participation interest over time. Future research could employ longitudinal designs or process-oriented data to further examine their developmental trajectories; (2) The sample was drawn from two universities in Jeollabuk-do, South Korea, which may limit the contextual generalizability of the findings. The applicability of the results across different cultural and educational settings requires further examination; (3) The study primarily relies on interview data and does not incorporate multiple data sources such as classroom observations or records of practical activities. Future studies could integrate multi-source data to enhance explanatory depth and validity; and (4) The study mainly focuses on micro-level interaction mechanisms within the learning process, with relatively limited attention to macro-level factors such as institutional environments and curriculum structures. Future research could adopt a multi-level perspective to explore how factors at different levels jointly influence the formation of sustainable experiential learning.

## 6 Conclusion

Grounded in the frameworks of Education for Sustainable Development (ESD) and Sustainable Development Goal 4.7 (SDG 4.7), this study employed a qualitative research design based on constructivist grounded theory to analyze dual-perspective interview data from tourism management teachers and students. The findings indicate that sustainable experiential learning in tourism management contexts does not arise from isolated practical activities. Rather, it is gradually constructed through the continuous interaction between teacher instructional guidance and student participation support, and is further transformed into stable participation mechanisms and psychological resources through processes of internalization. Specifically, (1) teacher instructional guidance, through structured guidance and reflective–emotional guidance, provides students with actionable pathways and supports their engagement under conditions of uncertainty; (2) student participation support, through cognitive coordination and emotion-regulatory support, facilitates experience sharing and the distribution of pressure, thereby sustaining the continuity of the learning process. On this basis, these forms of external support are gradually internalized through sustained participation: (a) manifested in the formation of psychological safety; (b) strengthened through practice-based experiences that enhance self-efficacy and activate participation interest; and (c) transformed into relatively stable positive psychological experiences.

Importantly, these psychological experiences function not only as learning outcomes but also as psychological resources that, in turn, sustain students’ continued engagement, making them more inclined to actively participate in subsequent learning activities. Therefore, sustainable experiential learning is not directly maintained by external arrangements. Instead, it emerges as a dynamic process in which support mechanisms are internalized into psychological resources, enabling learners to actively sustain their own participation over time.

Based on this, the present study provides a process-oriented explanation of the mechanism of “support–internalization–sustained engagement” in experiential learning within tourism management education. It clarifies the internal relationships among teacher instructional guidance, student participation support, and psychological processes, offering more explanatory empirical evidence for understanding students’ sustained engagement and professional development within the ESD framework.

## Data Availability

The original contributions presented in this study are included in the article/Supplementary material, further inquiries can be directed to the corresponding author.
