# Supplementary material for: From support to sustained engagement: internalization processes in experiential learning in tourism management education
Source: Front Psychol. 2026 May 14;17:1836705. doi: 10.3389/fpsyg.2026.1836705 (PMC13216208; doi:10.3389/fpsyg.2026.1836705)
Supplement: Supplementary file 1 [file Table_1.docx]

**Supplementary Table S1. Sampling Framework and Participant Characteristics**

| **Group** | **Sampling Dimension** | **Specific Criteria** | **Number** | **Analytical Purpose and Basic Information** |
| --- | --- | --- | --- | --- |
| Teacher Group  (N = 10) | Academic Field | Tourism management; hospitality management; cultural heritage tourism; tourism marketing (two comprehensive universities in Jeollabuk-do, South Korea) | 10 | Participants were embedded in diverse experiential teaching contexts, enabling cross-case comparison of instructional guidance practices. |
|  | Teaching Experience | Early-career (4–5 years); Mid-level (6–7 years); Senior (8–10 years) | 3 + 3 + 4 | Ensures variation in instructional design, uncertainty regulation strategies, and pedagogical decision-making across professional stages. |
|  | Experiential Teaching Practice | Field-based learning; destination project planning; service-learning; industry collaboration and internship supervision | – | Captures variation in structured guidance and reflective–emotional guidance across different experiential formats. |
| Student Group (N = 10) | Academic Year | Undergraduate Years 1–4 in tourism-related majors | 10 | All participants completed at least one in-depth experiential learning course. |
|  | Perceived Support Variation | Identified via screening interviews (high / medium / low perceived teacher guidance and peer support) | – | Ensures heterogeneity in support interpretation and internalization processes. |
|  | Engagement Trajectory Variation | Differences in participation interest, self-efficacy, and sustained engagement | – | Supports identification of dynamic psychological mechanisms across engagement stages. |

**Supplementary Table S2. Semi-Structured Interview Protocol**

| **Participant Group** | **Core Construct** | **Example Questions** |
| --- | --- | --- |
| Teacher Group | Structured Guidance | 1. How do you design and structure experiential learning tasks to help students navigate complex situations? |
|  | Reflective–Emotional Guidance | 2. When students encounter uncertainty or failure, how do you guide them to reflect and reinterpret these experiences? |
|  | Psychological Regulation | 3. How do you reduce students’ anxiety and help them sustain participation during challenging tasks? |
|  | Sustainable Learning Perspective | 4. What, in your view, constitutes a sustainable experiential learning environment? |
| Student Group | Teacher Instructional Guidance | 1. Can you describe how teacher guidance helped you understand tasks or reduce uncertainty? |
|  | Peer Support Mechanisms | 2. How did peers support you in task coordination or emotional regulation during projects? |
|  | Psychological Safety | 3. When did you feel safe to try, make mistakes, or express ideas? What contributed to that feeling? |
|  | Self-Efficacy & Interest | 4. Can you recall a moment when you felt capable or interested in continuing participation? |
|  | Sustained Engagement | 5. What motivates you to continue engaging in experiential learning over time? |

**Supplementary Table S3. Constructivist Grounded Theory Coding Procedures**

| **Coding Stage** | **Analytical Purpose** | **Procedures and Examples** | **Output** |
| --- | --- | --- | --- |
| Open Coding | Conceptualizing experiential processes | Line-by-line coding identified meaning units related to instructional guidance, peer interaction, uncertainty regulation, psychological safety, self-efficacy, participation interest, and sustained engagement. Initial codes remained close to participants’ language (e.g., “task decomposition,” “feeling safe to speak,” “shared responsibility,” “confidence after success”). | 92 initial concepts |
| Axial Coding | Linking support and psychological mechanisms | Codes were clustered through constant comparison to identify relationships between support types and psychological processes. Examples: Structured guidance (task scaffolding, clear criteria) Reflective–emotional guidance (risk reframing, meaning construction) Cognitive coordination (role complementarity, strategy sharing) Emotion-regulatory support (shared responsibility, reassurance) | 16 categories |
| Selective Coding | Building process-oriented model | Core category: Sustainable Experiential Learning Ecosystem. Process mechanism identified: External Support → Psychological Safety → Self-efficacy–Interest Cycle → Psychological Well-being → Sustained Engagement | 3 core themes + theoretical model |
| Trustworthiness | Ensuring rigor | Inter-coder agreement (30%), member checking, theoretical saturation (T9/S8), reflexive memos and audit trail | Credibility and transparency ensured |

**Supplementary Table S4. Theme 1: Dual-Pathway Structure of Teacher Instructional Guidance**

| **Main Category** | **Subcategory** | **Teacher Evidence** | **Student Evidence** |
| --- | --- | --- | --- |
| Structured Guidance | Task Structuring | “I divide the project into stages with clear requirements.” (T5) | “The structure helped me know what to do next.” (S8) |
|  | Process Transparency | “Students need clear criteria to evaluate their performance.” (T2) | “Once expectations were clear, I felt more confident.” (S4) |
| Reflective–Emotional Guidance | Risk Reframing | “I ask them what can be learned from failure.” (T7) | “It made me see failure as part of learning.” (S3) |
|  | Meaning Construction | “Growth matters more than perfect results.” (T9) | “That made me willing to try again.” (S6) |

**Supplementary Table S5. Theme 2: Interactive Coordination of Student Participation Support**

| **Main Category** | **Subcategory** | **Representative Evidence** |
| --- | --- | --- |
| Cognitive Coordination | Role Complementarity | “We divide tasks based on strengths.” (S2) |
|  | Strategy Sharing | “I learned methods from observing teammates.” (S7) |
| Emotion-Regulatory Support | Shared Responsibility | “We face problems together, not individually.” (S5) |
|  | Emotional Buffering | “Discussing with peers reduces anxiety.” (S6) |

**Supplementary Table S6. Internalization Pathways and Sustainable Engagement Process**

| **Stage** | **Mechanism** | **Qualitative Evidence** |
| --- | --- | --- |
| 1. Formation of Psychological Safety | External support reduces perceived risk and evaluation anxiety | Students reported feeling safe to try when teachers normalized failure and peers shared responsibility |
| 2. Self-Efficacy–Interest Reinforcement Cycle | Engagement → success → confidence → deeper engagement | Students described gaining confidence after task completion and becoming more interested in continued participation |
| 3. Psychological Well-Being Formation | Meaning, belonging, and controllability are stabilized | Students experienced learning as meaningful, socially supported, and manageable |
| 4. Sustained Engagement (Recursive Outcome) | Psychological resources feed back into future participation | Students expressed willingness to continue engaging in experiential learning beyond current tasks |
